# Professional Education

**Published:** 1900-02

**Authors:** 


A Monthly Review of Medicine and Surgery.

*EDITOR:*

WILLIAM WARREN POTTER, M. D.

All communications, whether of a literary or business nature, books for review and exchanges should be addressed to the editor: 284 FRANKLIN STREET, BUFFALO, N. Y.

VOL. XXXIX.—LV. FEBRUARY, 1900. No. 7.

### PROFESSIONAL EDUCATION.

A MOST instructive monograph has been written recently on the subject of professional education by Mr. James Russell Parsons, Jr., Director of the College and High School Departments, in the University of the State of New York, and since January 1, 1900, secretary, *ad interim*, of the University. This monograph is No. 10 of the series on education in the United States, edited by Professor Nicholas Murray Butler, of Columbia University, to be contributed to the United States educational exhibit by the State of New York at the Paris Exposition of 1900.

While the monograph was prepared primarily for those who propose to visit the exposition, as the author states, yet it is full of interest to all who are concerned in professional education. It shows in clear and concise manner the development of professional education in this country from the colonial period to the present, and is illustrated by a colored map and numerous diagrams. It tells students what they want to know in regard to the preliminaries required for entry into the professions, and it informs them what must be done to attain diplomas and licenses to practise after they have passed the collegiate entrance door.

The book deals with educational questions under the several heads of general education, theology, law, medicine, dentistry, pharmacy and veterinary medicine. All these several departments are considered in detail, their rise and progress set forth, their present status unfolded, their strength, standing and numerical attendance given, the libraries, endowments and plants they possess described, and their general financial condition recorded.

Of especial interest to physicians, however, is the section devoted to the consideration of medical education under the title, Medicine. It tells us that there were two medical schools in the colonies, the Medical College of Philadelphia (now the medical department of the University of Pennsylvania), organised in 1765, and the medical department of King's (now Columbia) College, organised in 1768. There are now 156 medical schools in the United States, of which 3 were established between 1765 and 1800, 12 between 1801 and 1825, 22 between 1826 and 1850, 33 between 1851 and 1875, and 86 between 1876 and 1900. In these 156 schools there were in attendance during the year 1899, 24,119 medical students, the increase in medical students in 21 years having been 142 per cent.

We are further told that for nearly a century there were practically no requirements in preliminary general education for admission to medical schools. In 1839 the Medical Society of the State of New York resolved that teaching and licensing ought to be separated as far as possible, and a similar view had been announced in Philadelphia two years before. In the development of higher standards New York passed a law that went into effect in 1891, providing certain preliminaries, extending the period of study in the schools, and establishing a final examination for license by a separate board. Under this law as since amended 4,808 physicians have been examined, of whom 3,722 were successful, or 77.5 per cent. If to them are added those who failed to meet the requirements for admission to the examinations, it will appear that more than 30 per cent. of all candidates have failed to secure licenses. When we consider that admission to the licensing examinations is predicated upon supposed preliminary education as well as a diploma from a duly registered medical school, the results seem somewhat startling; nevertheless, they furnish a complete justification of the system. A somewhat remarkable fact in relation to the subject is that the advanced requirments for license have been accompanied by a great increase in property holdings of the medical schools, whereas the reverse had been predicted and might have been at least temporarily expected.

One of the most perplexing questions that appears in relation to state license is interstate reciprocity, or recognition by endorsement. The National Confederation of State Medical Examiners has been studying, debating, and trying to solve the problem ever since it was organised, now some nine years ago. Lately the Wayne County (Michigan) Medical Society, probably impatient with the delay on the part of the Confederation, has sent circulars to licensing boards asking if reciprocity between states having equal standards would be desirable, and if, where such is not the case, statutory amendments would be advocated. This is like threshing over old straw, as the Confederation had done all this in its earlier inquiries. Of course, many favorable answers were received, but, as Mr. Parsons sententiously remarks, statutory amendments with few exceptions are found essential to establish the principle. Mr. Parsons further points out that a uniform standard for admission to practise in all the states is impracticable at present, because of varying conditions as to the density of population, educational advantages and general development. He states that weak states cannot establish standards demanded elsewhere and strong states cannot afford to lower their standards to meet the demands of the others. He, however, suggests as a remedy for the present needless and unfortunate multiplication of standards, that two or at most three standards be established for all the states, and that they be grouped according to their conditions. Naturally, under such a plan, in the first group would appear the states with the highest standards and these would act as a stimulus to the lower grade groups.

The State of Michigan only recently enacted a practice law, hence the zeal of youth in this matter of reciprocity can be easily understood, but before it proceeds much further in the premises it might be well for it to improve its record in one respect, for we are told by this monograph that it is one of the states in which osteopathy is recognised by law. As a matter of fact the first question of great importance relates to the establishment of a uniform minimum standard of preliminary education for admission to the study of medicine. When this is once agreed upon the problem of reciprocity of licensure will be more readily solved.

The section on medicine closes with a synopsis of present requirements to enter upon practice in all the states and territories, from which it appears, that in thirty-two diplomas do not confer the right to practise, a separate state examination being required of all; in five, the approval of medical diploma by duly qualified boards is sufficient; in nine, licensing examination only is required; while in fourteen political divisions, either approval of medical diploma or examination by independent boards are requisite. Alaska and Kansas have no examination boards.

This is a most interesting brochure, full of practical information and has been prepared with great care. It exhibits the hard work of an expert in all its sections, and will prove of great service to every educator. Besides, it will be a credit to the educational exhibit at Paris, for which it is more especially designed. Mr. Parsons is a graceful writer, a master of statistics, and an administrative officer of great accomplishment.

